# A Media Campaign to Increase Health Care Provider Assistance for Patients Who Smoke Cigarettes

**DOI:** 10.5888/pcd16.180613

**Published:** 2019-10-17

**Authors:** Harlan R. Juster, Christina A. Ortega-Peluso, Elizabeth M. Brown, Kim A. Hayes, Karla Sneegas, Gizelle Gopez, S. Rene Lavinghouze

**Affiliations:** 1 New York State Department of Health, Albany, New York; 2 RTI International, Research Triangle Park, North Carolina; 3 Office on Smoking and Health, National Center for Chronic Disease Prevention and Health Promotion, Centers for Disease Control and Prevention, Atlanta, Georgia

## Abstract

Although most smokers visit a health care provider annually, only half report being provided evidence-based assistance with quitting, defined as brief counseling and an offer of medication. The New York State Department of Health designed a provider-targeted media campaign to increase provider-assisted quitting, which was implemented in 2016. Messaging focused on the addictive nature of tobacco products and evidence-based interventions. Online surveys of 400 New York State health care providers measured advertising awareness, associations between awareness and assistance with quit attempts, and perceptions that patients expect providers to assist with quitting. Forty-three percent of providers were aware of at least 1 advertisement, and providers who had seen an advertisement were more likely to provide evidence-based assistance (AOR = 2.55, *P *= .01), which includes recommending or prescribing cessation medications. Provider-targeted media is a promising approach to reach health care providers and encourage evidence-based smoking cessation treatment.

**Summary**

**What is already known on this topic?**

Although most smokers visit a health care provider annually, only about half are offered evidence-based assistance in quitting. Counseling by a health care professional can at least double a smoker’s odds of successful quitting.

**What is added by this report?**

The New York State Department of Health designed a provider-targeted media campaign to increase provider-assisted quitting. Forty-three percent of providers were aware of at least 1 advertisement, and providers who had seen an advertisement were more likely to provide evidence-based assistance.

**What are the implications for public health?**

Reaching health care providers through targeted media can encourage evidence-based smoking cessation treatment.

## Introduction

Quitting nicotine addiction associated with cigarette smoking can be difficult (1,2). Clinical guidelines call for providers to ask about smoking status, advise smokers to quit, assess quit readiness, assist patients with brief counseling and medications, and arrange for follow-up care, referred to as the 5 A’s (Ask, Advise, Assess, Assist, and Arrange) (3). The New York State Department of Health (NYSDOH) has focused its tobacco control efforts on increasing cessation assistance by health care providers in the form of counseling (eg, education about the risks of smoking and the rewards of quitting) and the delivery of 1 or more US Food and Drug Administration (FDA)-approved smoking cessation medications. Delivery of counseling plus cessation medication can at least double the odds of successful cessation, and certain medication combinations further improve outcomes (3).

About 80% of cigarette smokers see a health care provider annually, making the potential reach of provider-based assistance greater than other recommended public health interventions (eg, telephone quitlines reach about 1% of smokers annually) (4). Although 90% of smokers report that their provider asked about tobacco use at their last visit (5), only 45% to 72% of patients who used tobacco were advised to quit, depending on patient demographics (6). Less than half of patients reported that their provider counseled them on cessation issues or recommended or wrote a prescription for medications (3,6). This is a missed opportunity to provide patients with treatment options that can ease withdrawal symptoms from nicotine and provide coping mechanisms to improve cessation outcomes. Providers cite as barriers to providing effective cessation treatments a lack of training, patients’ rejection or disinterest in quitting, and an assessment that other medical issues are more urgent than smoking (7), though effective counseling, such as motivational interviewing, can overcome patient barriers (2,8).

## Purpose and Objectives

NYSDOH developed, implemented, and evaluated a pilot project to determine the impact of a paid media campaign directly targeting providers with information about guideline-concordant, evidence-based smoking cessation strategies (3), including the most effective cessation methods, expected outcomes, and key prescribing information (Figure). This campaign complemented a broad public health strategy that included local NYSDOH contractors directed to engage with health system administrators in their catchment area to encourage adoption of system strategies from clinical guidelines (3) (eg, tobacco use screening in the electronic health record), and paid media directed at smokers to encourage quit attempts by engaging with providers. This broad set of strategies has the potential for reaching a large proportion of smokers with an evidence-based intervention, because 70% of smokers express a desire to quit (6) and 80% see a provider annually (4).

**Figure:**
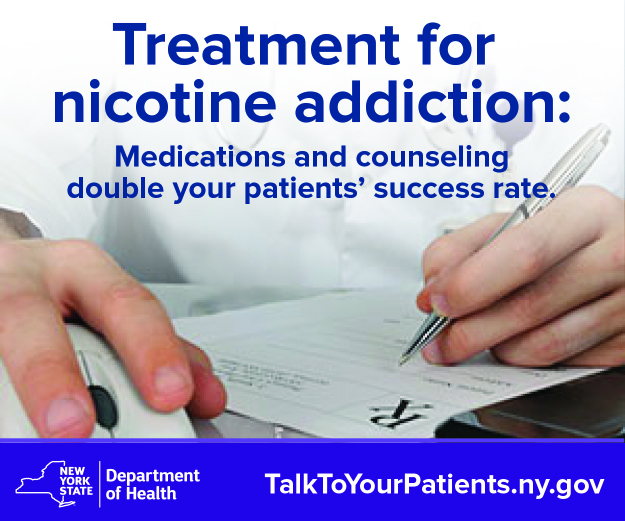

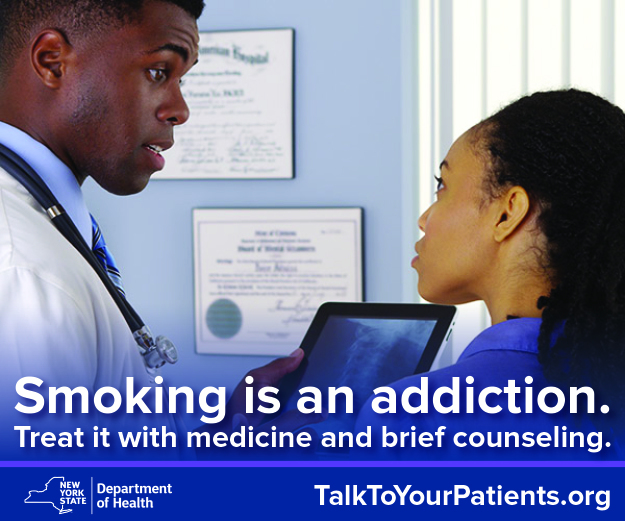

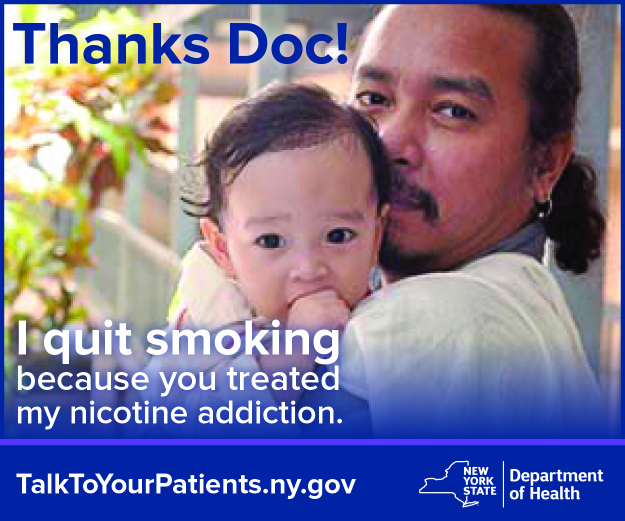
Three advertisements used in New York State Department of Health promotion of tobacco cessation patient interventions among health care providers. The 3 photographs provide links to a website: https://talktoyourpatients.health.ny.gov/.

The goals of this campaign were to determine whether a media strategy could reach primary care providers with messages they would find meaningful and would promote their use of evidence-based smoking cessation treatments. We hypothesized that campaign awareness would be positively associated with increased provision of cessation assistance. Evaluation measures reflected the core components of the campaign advertisements, including treatment-related beliefs and behaviors.

## Intervention Approach

Formative research was conducted in 3 phases, beginning with a review of evaluation studies conducted on behalf of NYSDOH to inform development of key campaign messages. Previous projects informed our ideas about provider attitudes toward patients who smoke, perceived barriers to treating smokers, and provider expectations about helping smokers quit. We examined patients’ awareness of available cessation insurance benefits (especially Medicaid) because lack of awareness can be a barrier to getting treatment.

Following this review, focus groups were conducted with physicians, nurse practitioners, and physician assistants. Participants were asked to discuss 4 message concepts: 1) addressing smoking as a critical part of care, 2) smoking cessation treatment does not take that much time, 3) if you don’t bring it up, patients might not think it’s important, and 4) quitting takes time and likely needs to be repeated to achieve success.

A recurring theme of the focus groups was the addictive nature of smoking, which providers believed warranted their intervention. The theme of addiction implied that quitting is difficult, that relapse is common, and that repeated treatment would likely be necessary. Addiction messaging was augmented with messaging regarding the need for evidence-based treatments, including specific actions providers should take: treat smoking and nicotine addiction with counseling and FDA-approved medication per clinical guidelines (3). As a result of the formative work, the concept of smoking as nicotine addiction was adopted as the central theme of the campaign (1).

In the third phase of the formative process, 3 advertisements were developed for testing among an online panel of providers. Testing assessed perceived effectiveness of advertisements, the extent to which advertisements educated providers about nicotine addiction and its treatment, the extent to which advertisements motivated them to help patients quit, and identification of advertisements most aligned with the intent of the provider-targeted media campaign. Advertisement placement was also guided by the results of the formative work, which indicated that providers primarily refer to social media and professional journals, both print and online, to access professional information. This was augmented with strategic out-of-home and hospital-based placement. Social media and online advertisements linked providers to a website developed by NYSDOH providing specific information about smoking cessation, counseling, and medications (https://talktoyourpatients.health.ny.gov/).

## Campaign Evaluation Methods

The media campaign ran from March through July 2016. In July, we surveyed 400 New York State health care providers recruited from Lightspeed Research’s online panel of providers. Lightspeed used email to recruit panel members who were physicians, nurse practitioners, or physician assistants in New York State, aiming for an even distribution across provider types. Providers were excluded if they did not provide patient care in the past 12 months or reported that 20% or fewer of their patients were adults. This was a convenience sample, and the online panel vendor did not report the number of providers invited to participate or share identifiable data on providers; the number of providers excluded is unavailable.

Evaluation measures reflected the core components of the campaign advertisements, including treatment-related beliefs and behaviors. The survey assessed advertisement awareness, provider use of evidence-based treatments for smoking cessation, provider perceptions of patient expectations, and provider demographics. For advertisement awareness, we showed providers each of the 3 advertisements and asked if they had seen the advertisements in the past 3 months. For provider perceptions of patient expectations, we instructed providers to indicate the extent to which they agreed or disagreed with the statement, “Patients expect that I should discuss tobacco use and quitting.”

We used previously established definitions for measures of ask, advise, and assist (9). Providers were asked how often in the past month they asked new or returning patients if they use tobacco. We categorized “always” or “often” as an affirmative response. We asked providers how often in the past month they advised patients who use tobacco to quit. For assistance, we asked how often in the past month providers did the following for patients who use tobacco: suggest that they set a specific date to stop using tobacco; suggest that they use a tobacco use cessation class, program, or counseling; suggest that they call a telephone quitline; provide them with booklets, videos, or other materials to help them quit on their own; and recommend or prescribe nicotine replacement or other stop-smoking medications when appropriate. We categorized assistance with a quit attempt as an “always” or “often” response to any of these 5 items.

We assessed provider characteristics of age, race/ethnicity, provider type, sex, cigarette smoking status, and cessation training in the past 5 years. We used 2 survey questions to classify smoking status as current smokers (smoked 100 cigarettes in their lifetime and currently smoke every day or some days), former smokers (smoked 100 cigarettes in their lifetime but currently do not smoke), and never smokers (have not smoked 100 cigarettes in their lifetime). For provider training, we asked whether providers participated in formal training or education on tobacco treatment and cessation counseling methods during the past 5 years.

We calculated post-stratification calibration weights using SUDAAN software’s PROC WTADJUST (10), based on the number of providers in New York State. We estimated descriptive statistics using Stata 14 (StataCorp LLC). We used adjusted Wald tests to assess the association between campaign awareness and provider beliefs about patients’ expectations about discussing tobacco use and quitting and between advertisement awareness and provider assistance. Logistic regression models were conducted to estimate adjusted odds ratios (AORs) with 95% confidence intervals (CIs) of the relationship between awareness of the campaign and key outcomes, controlling for covariates (age, race/ethnicity, provider type, sex, and training). The reference group for all logistic models were those providers not aware of the campaign. All study protocols were approved by RTI International’s institutional review board.

## Results

Respondents were physicians (33.5%), physician assistants (33.5%), and nurse practitioners (33.0%). Most respondents were female (59.5%) and white (81.0%). Overall, 20.8% had received tobacco-related training in the past 5 years. Most respondents were never cigarette smokers (74.8%); 22.5% were former smokers, and 2.8% were current smokers. The mean age of respondents was 47.6 (standard deviation, 12.0).

Forty-three percent of providers were aware of at least 1 of the advertisements. Campaign awareness did not differ by provider type. Advertisement awareness was associated with providers strongly agreeing that patients expected them to discuss smoking and quitting in bivariate analyses (*P* = .03), but not after controlling for other factors. The rate at which providers asked patients about tobacco use and advised them to quit did not differ by campaign awareness (Table). Providers aware of the campaign had greater odds of assisting tobacco users with a quit attempt than providers not aware of the campaign (AOR, 2.55; CI, 1.29–5.07; *P* = .01). Independent of other covariates, nurse practitioners had greater odds of assisting their patients than physicians (AOR, 4.05; CI, 1.45–11.3; *P* = .01).

**Table:** Provider (N = 400) Awareness of Provider-Targeted Media Campaign to Increase Patient Assistance With Tobacco Cessation, New York State Department of Health, 2016

Outcome	Aware of Campaign, % (95% CI) (n = 172)	Unaware of Campaign, % (95% CI) (n = 228)	*P* Value for the Difference	AOR (95% CI) [*P *Value]^a^
Strongly agree that patients expect providers to discuss tobacco use and quitting	25.4 (17.9–34.6)	14.1 (9.1–21.2)	.03	1.86 (0.95–3.65) [.07]
Ask patients about tobacco use	95.1 (88.4–98.0)	90.6 (84.0–94.6)	.19	1.81 (0.55–5.96) [.33]
Ask new patients about tobacco use	93.8 (86.6–97.2)	89.4 (82.6–93.7)	.24	1.80 (0.63–5.10) [.27]
Ask returning patients about tobacco use	80.1 (70.8–87.0)	73.1 (64.7–80.1)	.22	1.34 (0.67–2.68) [.41]
Advise patients to quit	92.1 (84.7–96.1)	84.4 (76.7–89.8)	.07	1.87 (0.74–4.72) [.19]
Assist patients with quitting	83.2 (74.2–89.5)	65.6 (55.8–72.6)	.001	2.55 (1.29–5.07) [.01]
Suggest setting a quit date	50.6 (41.0–60.1)	38.7 (30.7–47.3)	.07	1.45 (0.84–1.51) [.18]
Suggest a cessation class or program	63.3 (53.3–72.2)	45.6 (37.3–54.2)	.01	2.01 (1.15–3.53) [.02]
Suggest calling a quitline	43.5 (34.3–53.1)	16.9 (11.8–23.8)	<.001	3.84 (2.08–7.09) [<.001]
Provide self-help materials	37.1 (28.4–46.7)	16.4 (11.1–23.4)	<.01	2.80 (1.51–5.17) [<.001]
Prescribe or recommend nicotine replacement therapy or stop-smoking medications	55.3 (45.6–64.7)	41.5 (33.5–50.1)	.03	1.59 (0.92–2.75) [.10]

Abbreviations: AOR, adjusted odds ratio; CI, confidence interval.

a Providers aware of campaign versus providers not aware, estimated using logistic regression controlling for age, race/ethnicity, provider type, sex, and training. Reference group for all logistic regressions was providers not aware of the campaign.

## Implications for Public Health

Increasing smoking cessation rates is a public health priority (2). Although current population tobacco control interventions have produced lower cigarette smoking prevalence, declines have been slow (2,6). Opportunities exist to accelerate that decline if health care providers increase delivery of evidence-based cessation methods to their patients who smoke (6). As part of a comprehensive strategy to increase provider delivery of effective treatment in New York State (11), NYSDOH developed a media campaign targeting health care providers with messaging focused on the addictive nature of tobacco products and guideline-concordant treatment.

Evaluation of this campaign showed that providers can be reached by using print and digital media channels, with advertisements placed in medical journals and in and around hospitals. Moreover, messaging developed for this campaign was deemed meaningful by providers. Awareness of messages was associated with higher levels of evidence-based treatment delivery. Nurse practitioners showed the highest likelihood of providing cessation assistance, which may be related to their prominent role in current medical practice. This may suggest that future campaigns leverage messaging specific to nurse practitioners to enhance impact.

One limitation of our study is the cross-sectional nature of the survey; as a result, we are unable to causally attribute the outcomes of the study to the media campaign. Although the association of advertisement awareness with outcomes was possibly a function of the advertisements themselves, it is equally likely that provider differences in receptivity to the content increased the likelihood that providers would differentially attend to the advertisements. Providers already concerned about delivering effective cessation treatment might be more likely to notice the advertisements than providers who might not believe that providing cessation services is one of their primary responsibilities. Additionally, it is likely that these results are not necessarily representative of all health care providers in New York State because the sample came from a predetermined panel list and not a random sample. Reliance on self-reported data rather than objective behavioral measures is an additional limitation necessitated by the scope of the project.

This initial effort to reach health care providers with cessation-related messaging is encouraging. In response to this project’s limitations, we have identified a cohort of providers to follow longitudinally for the next campaign. Under these conditions, pre–post changes may be more indicative of exposure to campaign materials. Campaign messaging is also being modified on the basis of provider responses in the first phase and will focus more on directing providers to adopt specific treatment regimens shown to be most effective, especially combination medication therapies. Messaging is further being developed for a campaign focused on providers of behavioral health care. The results of this study are promising and suggest that a media campaign directed at health care providers with key messages focusing on a clear set of recommended treatment procedures could benefit the nearly 80% of smokers who see a provider annually. This approach for reaching a large proportion of smokers with effective provider interventions has far more potential than current public health standards (4).
